# 

**Published:** 1903-02

**Authors:** 


VOL. XVIII. FEBRUARY, 1903. No. 8.

### TEXAS
### Medical Journal.

SUBSCRIPTION, $1.00 A YEAR, IN ADVANCE.
SINGLE COPIES, 10 CENTS.

PUBLISHED AT AUSTIN, TEXAS, BY
F. E. DANIEL, M. D.,
PROPRIETOR.

Eastern Representative: John Guy Monihan, St. Paul Building, 220 Broadway, New York City.

Entered at the Postoffice at Austin, Texas, as Second-class Mail Matter.

